# Tumour Cell Labelling by Magnetic Nanoparticles with Determination of Intracellular Iron Content and Spatial Distribution of the Intracellular Iron

**DOI:** 10.3390/ijms14059111

**Published:** 2013-04-26

**Authors:** Zhigang Wang, Alfred Cuschieri

**Affiliations:** Institute for Medical Science and Technology, University of Dundee, Dundee DD2 1FD, UK

**Keywords:** magnetic nanoparticles, uperparamagnetic, SPIONs, nanomedicine, magnetic cell labelling, magnetic force microscopy

## Abstract

Magnetically labelled cells are used for *in vivo* cell tracking by MRI, used for the clinical translation of cell-base therapies. Studies involving magnetic labelled cells may include separation of labelled cells, targeted delivery and controlled release of drugs, contrast enhanced MRI and magnetic hyperthermia for the *in situ* ablation of tumours. Dextran-coated super-paramagnetic iron oxide (SPIO) ferumoxides are used clinically as an MR contrast agents primarily for hepatic imaging. The material is also widely used for *in vitro* cell labelling, as are other SPIO-based particles. Our results on the uptake by human cancer cell lines of ferumoxides indicate that electroporation in the presence of protamine sulphate (PS) results in rapid high uptake of SPIO nanoparticles (SPIONs) by parenchymal tumour cells without significant impairment of cell viability. Quantitative determination of cellular iron uptake performed by colorimetric assay is in agreement with data from the literature. These results on intracellular iron content together with the intracellular distribution of SPIONs by magnetic force microscopy (MFM) following *in vitro* uptake by parenchymal tumour cells confirm the potential of this technique for clinical tumour cell detection and destruction.

## 1. Introduction

All materials are magnetic to some extent, with their response depending on their atomic structure and temperature. They can be conveniently classified in terms of their volumetric magnetic susceptibility, χ, where *M =* χ*H* describes the (volumetric) magnetization *M* induced in a material by magnetic field (strength), *H*, with the material (relative) permeability, μ_r_, being defined as μ_r_ = (1 + χ). In SI units, χ is dimensionless, and both *M* and *H* are expressed in Am^−1^. Most materials display little magnetism and, even then, only in the presence of an applied field; these are classified either as paramagnets, for which χ falls in the range 10^−6^ to 10^−1^, or diamagnets, with χ being in the range −10^−6^ to −10^−3^. However, some materials exhibit ordered magnetic states and are magnetic even without an applied field. These are classified as ferromagnets, ferrimagnets and antiferromagnets, where the prefix refers to the nature of the coupling interaction between the electrons within the material. This coupling can give rise to large spontaneous magnetizations; in ferromagnets, *M* is typically 10^4^ times larger than would otherwise be the case.

The magnetic properties in ferromagnetic materials are the result of aligned unpaired electron spins. For these materials, magnetization is evident even in the absence of an external field. The transition between two magnetic domains (so-called Weiss domains) is referred to as a Bloch wall. At the nanometre scale (of the order of tens of nanometres or less, e.g., ~14 nm), the formation of Bloch walls becomes thermodynamically unfavourable, leading to the formation of single domain crystals, which are classified as superparamagnetic. The term superparamagnetic refers to the characteristic strong paramagnetic nature of the particles at this scale. Paramagnetic materials are distinguished by the tendency of their atomic magnetic dipoles to align with an external magnetic field, their small positive magnetic susceptibility (*i.e.*, ability to strengthen the field they are in) and their random orientation in the absence of a magnetic field (due to Brownian fluctuations). Superparamagnetic iron oxide (SPIO) nanoparticles have much larger susceptibilities (compared with strictly paramagnetic materials), as the entire crystal aligns with the applied field, due to its single crystal nature [1].

Magnetic nanoparticles offer a lot of attractive possibilities in biomedicine [2,3], and magnetic cell labelling is considered essential for the translation of cell-base therapies from the laboratory to clinical studies. Examples include magnetic separation of labelled cells and biological entities [4], targeted delivery and controlled release of drugs [5,6], magnetic hyperthermia for the *in situ* ablation of tumours [7] and contrast enhanced MRI [8–10]. Molecular and cellular magnetic resonance (MR) imaging is a rapidly growing field that aims to visualize targeted macromolecules or cells in living organisms by the use of superparamagnetic iron oxide (SPIO) nanoparticles (SPIONs) [1,11]. MR cell tracking, with its excellent spatial resolution, can be used as a non-invasive tool to provide unique information on the dynamics of cell movements *in vivo*. It is likely that MR cell tracking will be used in the future to monitor (stem) cell therapy in patients [12]. All these approaches require magnetic labelling of cells, as well as methods for analysis and evaluation of cell labelling [13,14]. Due to their biocompatibility and strong effects on T2* relaxation, iron oxide nanoparticles are now the MR contrast agent of choice for cell labelling [15], and several methods have been developed to incorporate sufficient quantities of iron oxide nanoparticles into cells [16,17].

A variety of cells have the natural ability to internalize exogenous material by a process known as endocytosis. The size of material and the rate at which it is internalized is dependent on the specific trans-membrane mechanism involved in the passage through the cell membrane. While specific cellular labelling by targeting specific binding sites on the surface of cells is possible, using SPIO nanoparticles coated with antibodies or other biological macromolecules, such as hormones or folic acid [18], our studies focused on non-specific cellular labelling. The majority of nonspecific cellular labelling has to date involved macrophages, as these cells are capable of efficient phagocytosis of exogenous/foreign particles, including micrometre-sized iron oxide particles [19]. In more general cases, the iron cellular uptake is not sufficient without the use of transfection agents or linking nanoparticles to the highly cationic HIV TAT peptide [20] or by using other physical means, such as magnetofection [21], electroporation [22] or sonoporation [23]. For example, cells have been successfully magnetically labelled by simply adding magneto-dendrimers, synthesized dendrimer encapsulated SPIOs [24], to the culture medium at concentrations of up to 25 μg iron/mL and incubation periods of one to two days.

For cell labelling application, magnetic nanoparticles have surface modification, usually by coating with biocompatible molecules, e.g., dextran, polyvinyl alcohol (PVA) and phospholipids. As well as providing a link between the particle and the target site on a cell or molecule, coating has the advantage of increasing the colloidal stability of the magnetic fluid. Ferumoxide (Feridex; Berlex, Wayne, NJ, USA) is a dextran-coated iron oxide [25], with particles sized between 80 and 120 nm. Ferumoxide is an FDA approved hepatic contrast agent, because the nanoparticles are spontaneously internalized by phagocytes (Kupffer cells). On post-contrast MRI scans, the phagocyte-rich liver turns dark, while the tumour, lacking macrophages, remains iso-intense (white). In our studies, for magnetic pre-labelling of non-phagocytic cells and cellular MRI, Ferumoxides have been combined with other commercially available transfection agents (TAs), such as poly-l-lysine (PLL) (Sigma, St. Louis, MO, USA) and Lipofectamine Plus (Invitrogen Life Technologies, Gaithersburg, MD, USA) [26].

We investigated several protocols for magnetic labelling of human cancer cells with ferumoxides [25] utilizing transfection agents, including poly-l-lysine (PLL), poly-l-ornithine (PLO) and protamine sulphate (PS), and applying electroporation [27]. In this report, we describe a quantitative assessment of magnetically labelled cells using conventional iron uptake measurement, as well as a magnetic force microscopy (MFM) study, which has the capability to resolve the spatial distribution of internalized magnetic nanoparticles [28–30] and can also be used to estimate the true uptake by a single cell and its resulting magnetization.

## 2. Results and Discussion

### 2.1. Magnetic Labelled Cells—Efficiency and Viability

**Figure 1:**
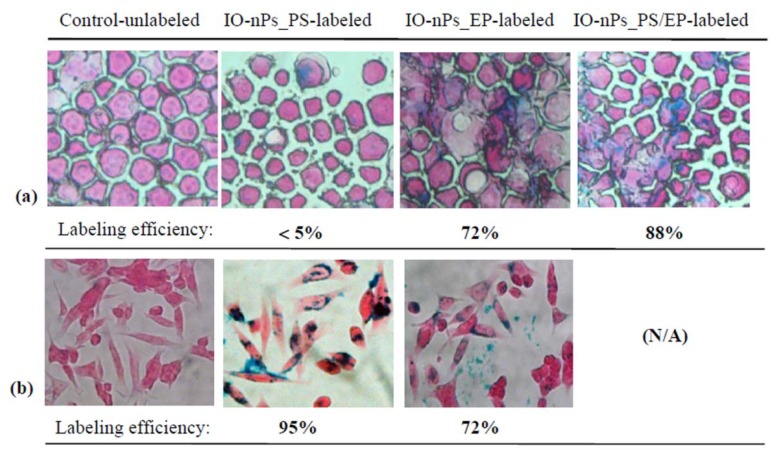
Cell labelling by electroporation and PS/electroporation: (**a**) stained promptly after electroporation (Cytospin staining); (**b**) stained after overnight culture. PS, protamine sulphate; EP, electroporation; IO-nPs, ferumoxides.

Cells were successfully labelled by magnetic nanoparticles (IO-nPs or ferumoxides) using Prussian blue staining, as illustrated (Figure 1). The resulting cell labelling efficiency and cell viability for three labelling methods used in this study are shown in Table 1. A very high labelling efficiency (95%) was obtained using protamine sulphate (PS) alone, while electroporation (EP) was only capable of loading cells with an efficiency of 72%. When PS was added to magnetic nanoparticles solution in electroporation mixtures, the loading efficiency increased from 72% to 88%.

With incubation of cells with PS, nanoparticle solution with PS did not significantly affect cell proliferation, but electroporation significantly decreased cell viability compared with controls, while combination (electroporation + PS) labelling significantly improved cell viability (Table 1). This combination labelling was also fast (*i.e.*, 30 min), obviating the need for prolonged incubation by transfection agents, *i.e.*, overnight (see Table 1 for comparison).

### 2.2. Assessment Cellular Iron Uptake and Spatial Distribution

**Figure 2:**
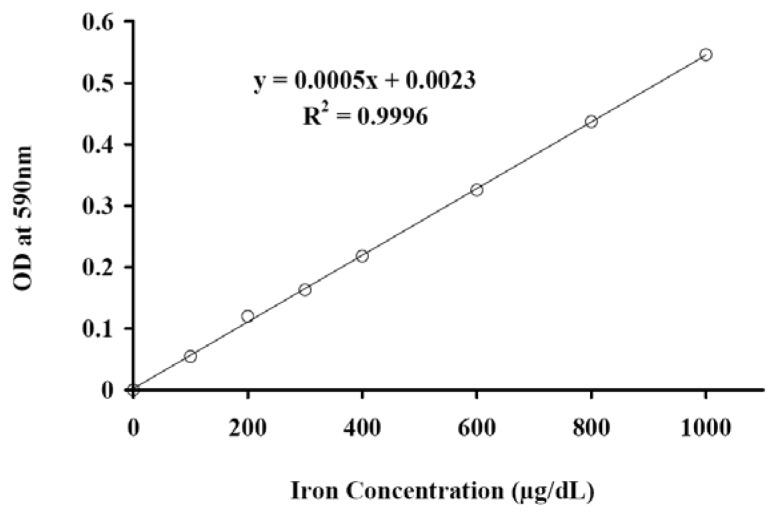
Standard curve in 96-well plate assay.

The standard optical density (OD) curve in a 96-well plate using the Quantichrom iron assay was plotted (Figure 2). This had a slope of 0.0005 with *R*^2^ = 0.9996. Using Equation (3) (described in Experimental Section), the amount of intracellular iron was estimated to be 3.773 ± 0.348 (*n* = 4) for the A375M cell line and 4.115 ± 0.564 (*n* = 4) for the MCF7 cell line (Table 2).

**Figure 3:**
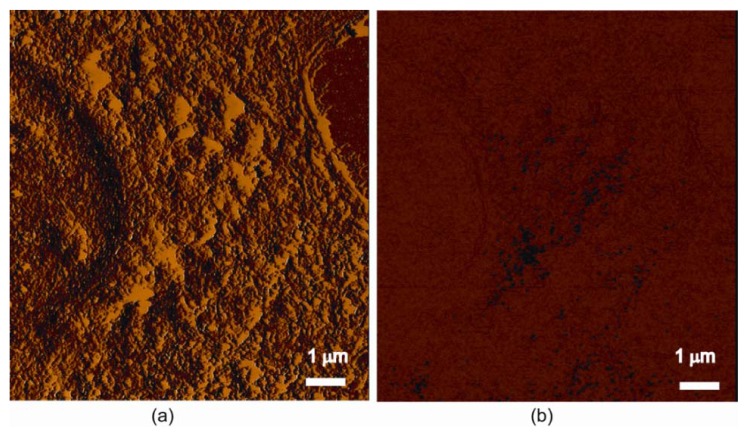

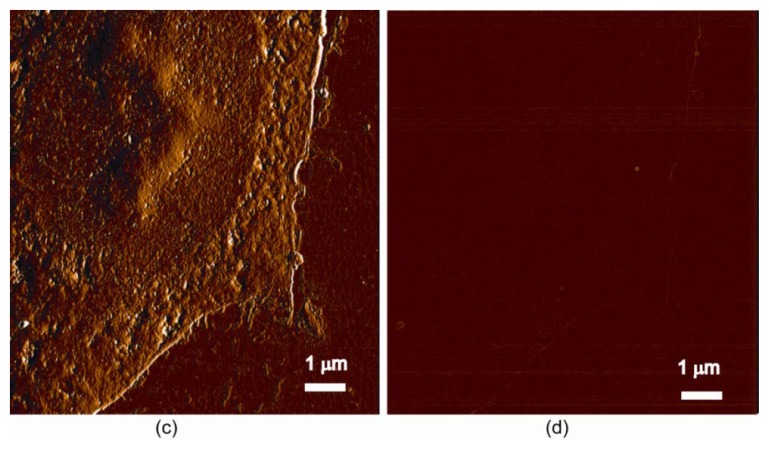
Magnetic force microscopy (MFM) images showing nanoparticles’ uptake and spatial distribution within single cells: (**a**–**b**) a labelled cell with morphological images in (**a**) and SPIOs uptake and a spatial distribution in phase (retrace) image (**b**); (**c**–**d**) an unlabelled cell for control with morphological images in (**c**), and no such phase shift detected in (**d**).

**Figure 4:**
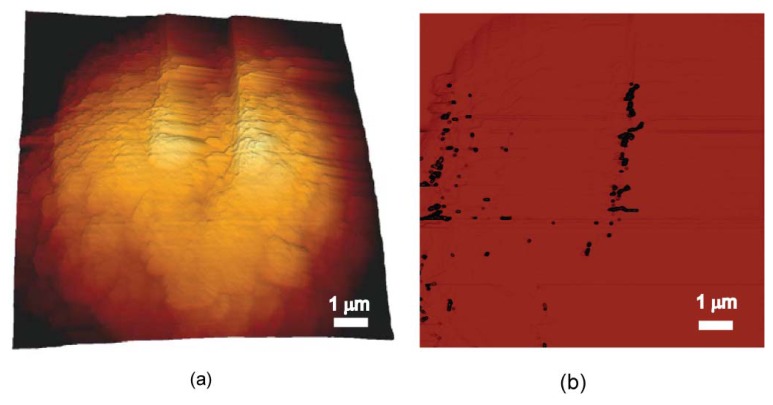
MFM images of a single cell: (**a**) 3D morphological image of the cell; (**b**) phase image in retrace mode (lift height of 100 nm from cell surface) showing SPIOs uptake and spatial distribution.

The spatial distribution of SPIOs following cellular uptake was demonstrated by stained optical images (Figure 1) and can be observed more closely in the accompanying MFM images (Figures 3 and 4). Cells can be observed in their morphological images in Figure 3a for a labelled cell and for an unlabelled (control) cell in Figure 3c. The uptake of SPIOs uptake is clearly shown in the phase (retrace) image (Figure 3b) for the labelled cell, while no such phase shift was detected in the control cell (Figure 3d).

SPIOs uptake by a single cell was observed using MFM (Figure 4), the quantitative iron uptake by the cell being estimated by Equation (5) (described in Experimental Section) at around 1.9 pg. The double-layer model provides an approximate iron uptake of 3.8 pg per cell.

### 2.3. Discussion

It is important to develop simple, accurate and low-cost methods for the determination of SPIOs iron for both clinical research and industrial work. The simplest iron determination method is the spectrophotometric one, which is based on protein precipitation, reduction of Fe^3+^ and formation of a coloured complex of Fe^2+^ with a chromogen [31]. The intensity of the colour, measured at 590 nm by spectrophotometry, colorimeter or a microplate reader being directly proportional to the iron concentration in the sample.

Iron content within *in vitro* labelled cells was evaluated using the Quantichrom iron assay. Wu *et al.* [32] reported that when 50 μL of samples containing 10^6^ cells were mixed with 200 μL Quantichrom Working Reagent in a 96-well plate (in triplicate) and incubated at room temperature overnight, the iron content per cell increased from 0.32 pg (protamine only) to 0.84 pg (ferumoxides) and 26.0 pg (Fe-Pro complex) 2 h after cell labelling.

The ferrozine-based assay is acknowledged as the most reliable method for colorimetric quantification of iron [22,24,26]. Ferrozine is an iron-chelating agent that forms a complex with ferrous iron (Fe^2+^) and exhibits characteristic UV-Vis absorption at 562 nm. The amount of intracellular iron as determined by the ferrozine-based colorimetric assay ranges between 1 and 5 pg Fe/cell, depending on the electroporation pulse conditions [22]. A more detailed description of colorimetric ferrozine-based assay can be found in [33]. This sensitive assay permits the quantification of iron in cultured cells in amounts ranging between 0.2 and 30 nmol. More importantly, estimates of cellular iron content obtained with the ferrozine-based assay are similar to those determined by the more expensive atomic absorption spectroscopy, MR relaxometry and radioactive detection, described below.

Atomic spectrometry: Inductively coupled plasma-atomic emission spectrometry (ICP-AES), also referred to as inductively coupled plasma optical emission spectrometry (ICP-OES), is a well-established technique for elemental analysis. For cellular iron uptake determination, the technique is based on the measurement of the emitted light of excited iron atoms. In the quantitative determination of intracellular iron uptake by human lung carcinoma cells (CLL-185) [8] by ICP-AES revealed a dose-dependent increase of iron oxide uptake (1.69 μg ± 0.11 Fe per 100,000 cells at 1.0 mg Fe/mL *vs.* 0.08 μg ± 0.01 Fe per 100,000 cells at 0.01 mg Fe/mL; *p* < 0.05) (Table 3). Inductively coupled plasma-mass spectrometry (ICP-MS) is an analytical technique that measures the mass-to-charge ratio of charged particles. Different chemicals have different masses, and this fact is used in mass spectrometry to identify chemicals present in a sample. The cellular uptake of micro-sized iron oxide particles analysed [19] by ICP-MS was reported to be 35 pg of Fe/cell (Table 3), which is consistent with results obtained by microscopy. Another clinically used paramagnetic contrast agents (apart from iron oxide based particles) is gadolinium (Gd)-based chelates, and the intracellular concentration of Gd per cell can also be measured using ICP-MS [34].

Magnetic resonance (MR) relaxometry: MR relaxometric methods are also used to measure the concentration of iron. They are based on the linear relationship between iron content and MR relaxation rates of 1/T1 or 1/T2 [24,26]. By using either custom designed equipment or commercially available MRI scanners, T1 and T2 relaxation rates obtained from samples are compared with the known iron concentration in serial dilutions of iron solution that is used for generating standard calibration curve. Bulte *et al*. [24] estimated cellular uptakes of 9.3 ± 4.3 and 13.6 ± 5.5 pg iron/cell for the CG-4 (rat oligodendrocyte progenitor) and HeLa (human cervix carcinoma) cells, respectively. This was in agreement with the corresponding values obtained using the Ferrozine assay, which was 8.5 ± 2.0 and 13.6 ± 2.9, respectively (Table 3).

Radioactive detection: In this technique, Dextran-coated SPIO are modified with diethylenetriamine penta-acetic acid (DTPA) for isotope labelling, *i.e.*, using a radiotracer (^111^In). The radiolabeled cross-linked iron oxide (CLIO)-TAT particles, counted with a gamma counter, result in labelling efficiencies ranging [20] from 10 to 30 pg of superparamagnetic iron per cell (Table 3). Our *in vitro* study suggests that electroporation in the presence of protamine sulphate (PS) as a transfection agent can provide an effective and fast technique for labelling various types of cells with magnetic nanoparticles and provides a method that can be used clinically. Our quantitative results obtained by the Quantichrom iron assay (3.8 to 4.1 pg per cell) are in good agreement with the values in the published literature (*i.e.*, from 0.8 to 35 pg/cell, Table 3), depending on cell types, SPIOs size and concentration, surface modification, incubation time, *etc.* The MFM imaging data demonstrate the spatial distribution of internalized IO-nPs. Furthermore, with the simplified geometrical model, the MFM-based single cell imaging assessment of IO-nPs’ uptake can provide additional quantitative information on the individual magnetized cell (1.9 to 3.8 pg). More advanced modelling and simulation would provide more accurate quantitative information of single-cell magnetization based on MFM scanned image and other imaging modality, such as TEM, to estimate SPIOs’ aggregation depth inside the cell [30].

Finally, magnetofection is a relatively new transfection method and is achieved by the application of a magnetic field to superparamagnetic iron oxide particles [35]. Magnetofection exploits the magnetic forces that guide the SPIOs, associated with gene vectors, DNA plasmids, toward the target cells. The presence of these magnetic fields increases the transfection efficiency, compared to cells not exposed to the magnetic field [35–39]. Further investigation with magnetic nanoparticles coated with positively charged groups [40] for more cell labelling experiments, especially with the used of the our recently reported magnetoporation method [41] by an low-intensity external rotating magnetic field, is underway, since magnetoporation/magnetofection have potential over electroporation in clinical applications.

## 3. Experimental Section

### 3.1. Cell Labelling by Magnetic Nanoparticles

We investigated different magnetic cell labelling methods using magnetic nanoparticles (SPIOs or IO-nPs), including transfection agents and electroporation. Our aim was to identify an effective and fast technique for potential clinical use. The work was based on the hypothesis that SPIOs, coated and mixed as complexes with cationic transfection agents, can be more effectively aligned to cell membranes, as these are slightly negatively charged, thereby achieving more efficient labelling through trans-membrane passage by electropermeabilization and other mechanisms, including natural endocytosis. Several human cancer cell lines (A375M, DLD1, MCF7, SW480 and U2OS) were used for the labelling studies.

#### 3.1.1. Labelling with Transfection Agent (PS)

The transfection agent, protamine sulphate (PS), at varying concentrations was mixed with IO-nPs (60 μg/mL) in culture media for 15 min at room temperature with intermittent hand shakings. The culture medium containing the transfection agent-IO-nPs complexes were added to the cell cultures such that the final concentration of IO-nPs was 30 μg/mL, and the final concentrations of PS ranged between 1.0–5.0 μg/mL in cell viability experiments and 3.0 μg/mL for other experiments. Cells were then incubated overnight, for approximately 16–18 h.

#### 3.1.2. Labelling with Electroporation

Electroporation was performed with a Nucleofector Device II from Amaxa Biosystems GmbH (Cologne, Germany), according to the manufacturer’s instructions. Cells suspended in Nucleofector Solutions were mixed with IO-nPs media before transfer to electroporation cuvettes. Specific intensities and lengths of electric pulse were selected and used to obtain optimal labelling. Control experiments were performed by processing cells in the same way, but in the absence of IO-nPs. After electroporation, cell suspensions were diluted with pre-warmed complete DMEM and transferred to culture plates. The final concentration of IO-nPs in culture medium was 100 μg/mL. Cells were incubated overnight or immediately assessed for instant labelling or cell viability.

#### 3.1.3. Labelling with Electroporation in the Presence of PS

This was carried out by the same procedure and addition of PS-IO-nPs complexes to the pre-warmed medium used to dilute the electroporation mixtures. In order to maintain comparable conditions, the final concentrations were kept at 3 μg/mL and 100 μg/mL for PS and IO-nPs, respectively.

### 3.2. Assessment of Magnetically Labelled Cells

#### 3.2.1. Cell Viability by MTS Assay

The MTS Cell Titre 96 AQueous One Solution Cell Proliferation Assay (Promega, Southampton, UK) was used to determine cell viability in control and treated cell studies. Cells were seeded at densities of 5000–10,000 cells/well in 96-well plates and were either assayed at 30 min (after electroporation) or left to adhere for 16–18 h for transfection treatments before MTS assay. The MTS assay values (absorbance at 490 nm) were expressed as the percentage of that of the corresponding control cells.

#### 3.2.2. Prussian Blue Staining for Assessing Labelling Efficiency

Cells grown on glass coverslips were washed with PBS to remove excess of IO-nPs and fixed with 4% para-formaldehyde for 30 min. They were then washed with ddH2O and incubated for 30 min with 2.5% potassium ferrocyanide in 2.5% HCl following counterstaining with nuclear fast red for 5 min. The samples were examined using a Zeiss microscope Axiovert 200 (Zeiss, Oberkochen, Germany) at ×40 and ×63 magnification and Axiovision 4.6 software. Cells were considered Prussian Blue positive if intra-cytoplasmic blue granules could be identified. The efficiency of cell labelling was determined by manual counting of Prussian Blue-positive and -negative cells. The percentage of labelled cells was determined from the average of 5 to 10 fields (×40).

#### 3.2.3. Quantitative Colorimetric Iron Assay

The Quantichrom iron assay (BioAssay Systems, Hayward, CA, USA), a quantitative colorimetric iron determination at 590 nm, was used for studying cellular iron uptake. In accordance with the manufacturer’s protocol, 50 μL of standards or samples containing 10^6^ cells were mixed with 200 μL Quantichrom Working Reagent in a 96-well plate and incubated at room temperature overnight [32]. In this system, the optical density (OD) at 590 nm measured by a microplate reader is directly proportional to the iron concentration in the sample. The OD against standard iron concentrations were plotted by subtracting blank (water) OD from the standard OD values, and the slope of the data plot then determined using liner regression fitting (Slope = 0.0005, see Figure 3). The iron concentration (IC_sample_ in μg/dL) of the labelled sample can be calculated as:

(1) $${\text{IC}}_{\text{sample}}=\frac{{\text{OD}}_{\text{sample}}-{\text{OD}}_{\text{blank}}}{\text{Slope}}$$

where OD_sample_ and OD_blank_ are OD_590nm_ values of the labelled cell suspension sample and the blank water sample. Given cell concentration (CC_sample_) in the labelled cell suspension sample of *N* (cell/dL) (*N* = 10^6^ cells/(50 μL + 200 μL) in this assay), averaged iron uptake per cell (iron uptake in pg/cell) can be obtained from:

(2) $$\text{Iron Uptake}=\frac{{\text{IC}}_{\text{sample}}}{{\text{CC}}_{\text{sample}}}=\frac{{\text{OD}}_{\text{sample}}-{\text{OD}}_{\text{blank}}}{\text{Slope}}\frac{1}{N}10^{6}$$

### 3.3. Magnetic Force Microscopy (MFM) Observation and Assessment

#### 3.3.1. Magnetic Force Microscopy (MFM) Principle

MFM is a special mode of operation of the non-contact scanning atomic force microscope (AFM), with high (~25–50 nm) spatial resolution [28,29]. The principle of MFM is to measure the change of the interaction force (*F*_m_) between a magnetized probe (of strength *H*) and the local stray magnetic field (induction), *B*, from the sample as the probe is scanned across the surface in dynamic (non-contact) mode by :

(3) $$F_{m}=(m\cdot \nabla )B$$

where *m* = *MV* is the magnetic moment on a volume, *V*, of the material with magnetisation of *M* (*M* = χ*H* and χ is material’s volumetric magnetic susceptibility, which can be obtained SPIOs’ material magnetization curve), and each individual atomic moments in the material contribute to its overall magnetic induction (*B*) response as:

(4) $$B=\mu_{0}(H+M)$$

where μ_0_ is the permeability of free space.

One standard method of obtaining a MFM image is to operate the AFM in close-contact mode with a magnetic cantilever that detects a force gradient, which contains information from both the surface structure and the local magnetic field. After collecting a topographic image close to the surface, the cantilever is then ‘raised’ some height above the surface, where the magnetic forces dominate on the reverse scan. Signals from surface topography dominate at close distances to the surface, while at greater distances from the surface, the magnetic signal dominates. Consequently, depending on the distance between the surface and the tip, normal MFM images may contain a combination of topographic and magnetic signals. Standard magnetic recording tape was used to obtain known magnetic domain pattern image for assisting selection of the MFM tip lift height (e.g., 100 nm in this study).

#### 3.3.2. MFM Scans with Image Processing for Single Cell Assessment

IO-nPs labelled cells (SW480) were prepared, and the air dried glass slides of samples were used for MFM. Cell uptake was imaged using a JPK Nanowizard atomic-force microscope (AFM) (JPK Instruments, Berlin, Germany) on top of an Axiovert 200 inverted microscope (Carl Zeiss, Jena). MFM imaging using phase detection was performed in hover (lift) mode within intermittent contact (dynamic) mode. The lift height was 100 nm distance between the cantilever tip and the surface of the sample, and the MFM scan was performed at a scan rate of 1 Hz with a resolution of 512 × 512 pixels. The MFM cantilevers were made of silicon coated with cobalt-chromium alloy (Hc = 300–400 Oe, spring constant: 1–5 N/m, MFMR-10, Nanosensors Inc., Darmstadt, Germany).

MFM scan image of SPIOs uptake with a complete single cell was then further processed by a custom-written Matlab (MathWorks, Cambridge, UK) codes for imaging analysis to obtain a total SPIOs’ area *A*_SPIOs_. To assess the SPIOs’ uptake within the image, a simplified SPIO single-layer geometrical model was used to obtain the SPIOs’ volume *V* (*V* = *A*_SPIOs_ × *D*_SPIO_, where *D*_SPIO_ is the diameter of a single SPIO particle). Given a volumetric density, *ρ*, of the used SPIOs, we could estimate the mass *M*_SPIOs_ of the uptake SPIOs by:

(5) $$M_{\text{SPIOs}}=\rho V$$

where *ρ* = 6000 kg/m^3^ [42]. A double-layer model can also be used to estimate SPIOs’ aggregation inside the cell [30].

## 4. Conclusions

This *in vitro* study indicates that electroporation in the presence of the clinically used transfection agent, protamine sulphate (PS), can provide an effective and fast technique for labelling of various cell types with magnetic nanoparticles (e.g., ferumoxides, a clinical proved SPIOs). The Quantichrom iron assay provides a simpler method for iron determination *in vitro*, compared to atomic spectrometry, MR relaxometry and radioactive detection. The MFM scan provides an unique method for observing any (not just iron-based) cellular uptake of magnetic particles ranging from the nano-meter up to 100 micro-meter scale, and further quantitative information of individual cells can be derived from image processing using simple or complex models.

Electro-magnetoporation can provide an efficient tool for potential *in situ* clinical use by which *in vivo* quantification of cell/tissue iron uptake can be obtained by MR relaxometry in addition to tracking of the labelled cells by MRI.

## Figures and Tables

**Table 1:** Comparison of cell labelling by transfection, electroporation and PS/electroporation (PS, protamine sulphate; EP, electroporation; IO-nPs, ferumoxides).

Labelling method	PS	EP	PS/EP
Final concentration of IO-nPs (μg/mL)	30	100	100
Final concentration of PS (μg/mL)	3.0	–	3.0
Duration of procedure	14–16 h	30 min	30 min
Labelling efficiency (%)	95	72	88
Cell viability (A 375M)	98.73 ± 5.56	73.21 ± 7.21	89.34 ± 3.56

**Table 2:** Quantitative cellular iron uptake: labelled with IO-nPs (ferumoxides) using PS/EP and measured by the Quantichrom iron assay (PS, protamine sulphate; EP, electroporation).

Iron uptake	Labelled (pg/cell)	Control (pg/cell)
A375M (melanoma)	3.773 ± 0.348 (*n* = 4)	0.075 ± 0.130 (*n* = 4)
MCF7 (breast)	4.115 ± 0.564 (*n* = 4)	0.179 ± 0.229 (*n* = 4)

**Table 3:** Reported cellular uptake rates of iron.

Measurement	Uptake (pg Fe/cell)	Cells #	Magnetic particles & labelling *	References
ICP-AES	16.9 ± 1.1	CLL-185	SPIOs (1 mg/mL) and lipofection	[8]
ICP-AES	0.8 ± 0.1	CLL-185	SPIOs (10 μg/mL) and lipofection	[8]
ICP-MS	35	B16F10	MPIO beads and macrophages	[19]
MR relaxometry	9.3 ± 4.3	CG-4	SPIOs (1–25 μg/mL) and dendrimers	[24]
Ferrozine assay	8.5 ± 2.0	CG-4	SPIOs (1–25 μg/mL) and dendrimers	[24]
MR relaxometry	13.6 ± 5.5	HeLa	SPIOs (1–25 μg/mL) and dendrimers	[24]
Ferrozine assay	13.6 ± 2.9	HeLa	SPIOs (1–25 μg/mL) and dendrimers	[24]
Relaxometry/Ferrozine	3.8 ± 1.2	CG-4	Ferumoxides and PLL (25 μg/mL)	[26]
Gamma counter and ^111^In	10 to 30	CD34^+^	CLIO-Tat peptides (100 μg/mL)	[20]
Ferrozine assay	1 to 5	NSC (C17.2)	Ferumoxides (2 mg/mL) and EP	[22]
Quantichrom assay	26.0	Leukocytes	Ferumoxides (50 μg/mL) and PS	[32]

# Cell lines used in *in vitro* measurement—B16F10: melanoma; CLL-185: human lung carcinoma cells; CG-4: rat oligodendrocyte progenitor; HeLa: human cervix carcinoma; CD34^+^: human hematopoietic cells; MSC: mesenchymal stem cells; NSC (C17.2): neural stem cells.

* Labelling method—EP: electroporation; PS: protamine sulphate; ICP-AES, inductively coupled plasma-atomic emission spectrometry; ICP-MS, inductively coupled plasma-mass spectrometry.
